# Potential and active functions in the gut microbiota of a healthy human cohort

**DOI:** 10.1186/s40168-017-0293-3

**Published:** 2017-07-14

**Authors:** Alessandro Tanca, Marcello Abbondio, Antonio Palomba, Cristina Fraumene, Valeria Manghina, Francesco Cucca, Edoardo Fiorillo, Sergio Uzzau

**Affiliations:** 1 Porto Conte Ricerche, Science and Technology Park of Sardinia, S.P. 55 Porto Conte - Capo Caccia km 8,400, Località Tramariglio, 07041 Alghero, SS Italy; 2 Department of Biomedical Sciences, University of Sassari, Sassari, Italy; 3 Istituto di Ricerca Genetica e Biomedica, Consiglio Nazionale delle Ricerche (CNR), Monserrato, Cagliari, Italy

**Keywords:** Gut microbiota, Metagenomics, Metaproteomics, Metabolic pathway, *Faecalibacterium*, Short-chain fatty acids

## Abstract

**Background:**

The study of the gut microbiota (GM) is rapidly moving towards its functional characterization by means of shotgun meta-omics. In this context, there is still no consensus on which microbial functions are consistently and constitutively expressed in the human gut in physiological conditions. Here, we selected a cohort of 15 healthy subjects from a native and highly monitored Sardinian population and analyzed their GMs using shotgun metaproteomics, with the aim of investigating GM functions actually expressed in a healthy human population. In addition, shotgun metagenomics was employed to reveal GM functional potential and to compare metagenome and metaproteome profiles in a combined taxonomic and functional fashion.

**Results:**

Metagenomic and metaproteomic data concerning the taxonomic structure of the GM under study were globally comparable. On the contrary, a considerable divergence between genetic potential and functional activity of the human healthy GM was observed, with the metaproteome displaying a higher plasticity, compared to the lower inter-individual variability of metagenome profiles. The taxon-specific contribution to functional activities and metabolic tasks was also examined, giving insights into the peculiar role of several GM members in carbohydrate metabolism (including polysaccharide degradation, glycan transport, glycolysis, and short-chain fatty acid production). Noteworthy, Firmicutes-driven butyrogenesis (mainly due to *Faecalibacterium* spp.) was shown to be the metabolic activity with the highest expression rate and the lowest inter-individual variability in the study cohort, in line with the previously reported importance of the biosynthesis of this microbial product for the gut homeostasis.

**Conclusions:**

Our results provide detailed and taxon-specific information regarding functions and pathways actively working in a healthy GM. The reported discrepancy between expressed functions and functional potential suggests that caution should be used before drawing functional conclusions from metagenomic data, further supporting metaproteomics as a fundamental approach to characterize the human GM metabolic functions and activities.

**Electronic supplementary material:**

The online version of this article (doi:10.1186/s40168-017-0293-3) contains supplementary material, which is available to authorized users.

## Background

In the latest years, the study of the gut microbiota (GM) has been shifting from a mere description of the taxonomic composition, usually obtained through the application of 16S rRNA gene sequencing to fecal samples, to a broader investigation of GM functional potential, made possible by shotgun metagenomics (MG) approaches [1]. Population MG studies have revealed that GMs share a stable set of core functions, in spite of a large inter-individual structural/compositional variability [2, 3]. However, since sequenced genes are not necessarily expressed [4, 5], MG cannot provide reliable information on which microbial functional traits are actually changing in response to stimuli from host metabolism, immunity, neurobiology, diet, or other environmental factors. Conversely, this type of information can be gathered by functional meta-omics, as metatranscriptomics (MT) and metaproteomics (MP), which display higher sensitivity to perturbation and may therefore better reflect host-microbiome interactions [6]. In this context, of particular interest is to investigate the relationship between potential and actually active GM features in a human population, in order to identify microbial functions constitutively expressed in a healthy gut starting from a known MG potential. A recent investigation has addressed this aim with respect to MT, finding transcripts of ribosomal proteins and citrate cycle enzymes among those with the highest expression rate (mRNA/DNA ratio) and genes involved in starch metabolism, amino acid biosynthesis, sporulation, and peptidoglycan biosynthesis as those with the lowest expression rate [7]. Less is known about microbial proteins, even though these provide major information concerning GM metabolism and represent key molecules in the host-GM interaction. Although a few pioneering studies have presented the analysis of paired metagenomes and metaproteomes in disease-related human cohorts [6, 8], a systematic, comparative investigation of taxonomic and functional features potentially and actually expressed by the GM of a healthy population has not been described so far.

Here, we selected a cohort of healthy subjects from a clinically monitored Sardinian population and collected from each subject a stool sample which underwent DNA and protein extraction, followed by shotgun MG and MP analyses. MG and MP data were then mined in a comparative fashion in order to (i) find which functional features are actively and consistently expressed by the GM, being therefore needful for the host-GM homeostasis; (ii) identify conserved and variable GM features within the population; and (iii) investigate the specific functional and metabolic contribution of the key GM taxa.

## Results

### Experimental design and general metrics

Fifteen subjects were selected from the SardiNIA study cohort [9]. Stool samples were collected from individuals self-reporting the absence of (i) antimicrobial treatment during the previous 6 months from sample collection, (ii) inflammatory bowel disease and other autoimmune conditions, (iii) significant variations of body temperature during the last 2 weeks, and (iv) unusual body weight fluctuation during the last 3 years before sample collection. Subjects were selected to avoid sex, age, and body mass index (BMI) biases (Additional file 1: Table S1), and all followed an omnivorous diet.

**Fig. 1:**
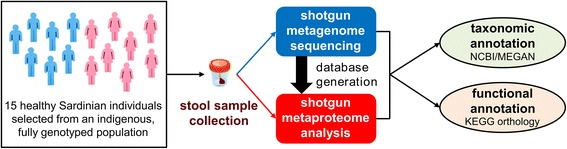
Schematic illustrating the experimental design of the study. Fifteen healthy adult subjects (7 males and 8 females) were selected from a clinically monitored Sardinian population. Stool samples were collected from each individual and subjected in parallel to Illumina shotgun DNA sequencing (metagenome profiling) and LTQ-Orbitrap shotgun mass spectrometry analysis (metaproteome profiling). The metagenomes were also employed as sequences databases, in order to allow a rigorous metaproteome/metagenome comparison, and subjected to taxonomic and functional annotation

As illustrated in Fig. 1, a stool sample was collected from each subject, and its metagenome and metaproteome were characterized by means of shotgun MG and shotgun MP, respectively. A population-based matched database, comprising all MG sequences retrieved from the same cohort under study, was used for MP analysis in order to map protein expression of the very same genes identified by MG. MG sequences were annotated both taxonomically and functionally, and these two annotation levels were linked to address the question on “who is doing what” within the GM of the selected subjects.

A total of 25,993,645 MG reads and 107,069 peptide-spectrum matches (PSMs) were obtained in this study, with a mean of 2,077,370 reads and 7138 PSMs per sample (Additional file 1: Table S2). In view of the inter-individual variability in the total number of reads, MG reads were randomly subsampled to allow a better comparison among samples. Taxonomic and functional annotation yields varied between MG and MP, and the relative amount of reads/peptides assigned to a specific genus varied between Firmicutes and Bacteroidetes (Additional file 1: Table S3). Globally speaking, MG data exhibited a larger depth of information compared to MP, both in taxonomic and functional terms, as expected and previously reported [6, 8].

### Potential and active functions in the gut microbiota

**Fig. 2:**
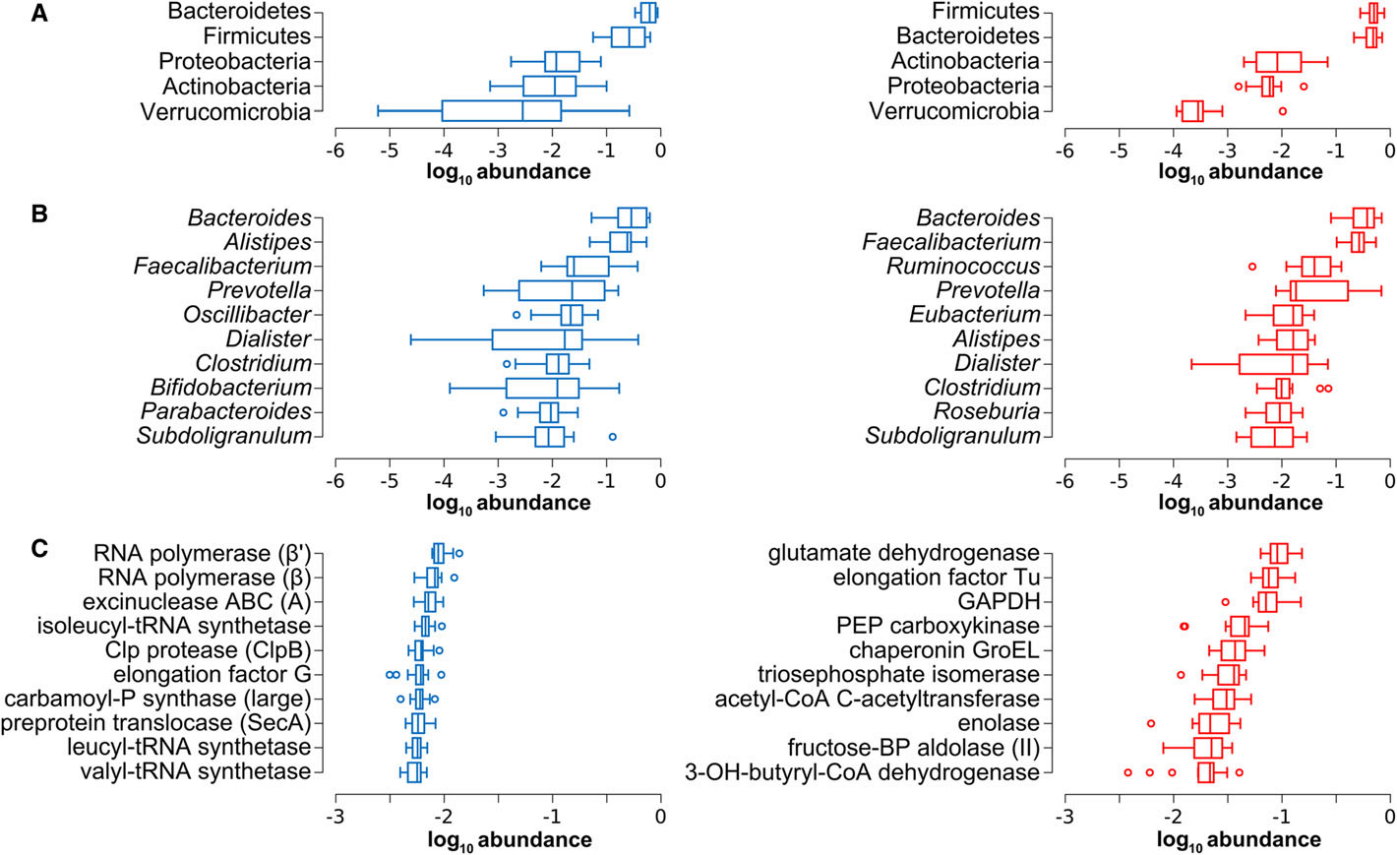
Main metagenome and metaproteome features of the gut microbiota. MG data are in *blue* (*left*), while MP data are in *red* (*right*). Data are ordered by decreasing median of the relative abundance among subjects. **a** Tukey’s boxplots showing the top 5 microbial phyla. **b** Tukey’s boxplots showing the top 10 microbial genera. **c** Tukey’s boxplots showing the top 10 gene/protein functions (KEGG orthology groups). Subunit names are shown into brackets. *GAPDH* glyceraldehyde 3-phosphate dehydrogenase, *PEP* phosphoenolpyruvate, *P* phosphate, *BP* bisphosphate, *OH* hydroxy

A preliminary, unsupervised multivariate analysis revealed a much clearer separation between MG and MP patterns based on functional data when compared to taxonomic data (Additional file 2: Figure S1). The most abundant phyla (A), genera (B), and functions (KEGG orthologous groups (KOGs); panel C) detected by MG and MP are illustrated in Fig. 2. Consistently, a large overlap could be observed between MG and MP regarding the most abundant phyla and genera, in contrast with considerable differences in functions, highlighting a divergence between functional potential and activity. In particular, enzymes belonging to catabolic pathways were generally massively abundant, while not being among the genes present with the highest number of copies in the metagenome. Furthermore, correlation between MG and MP profiles was high when considering taxa abundances, with a linear decrease when going down to lower taxonomic levels (Spearman’s *ρ* = 0.90 ± 0.06 (mean ± s.d.) at phylum level, *ρ* = 0.68 ± 0.07 at genus level), while a considerably lower correlation could be found for functional features (*ρ* = 0.21 ± 0.06).

We then carried out a comparative investigation of MG and MP features, in order to identify GM functions consistently expressed within the healthy human cohort under study, taking into account the gene potential of the same GMs. To this aim, we computed the log MP/MG abundance ratio for each taxon and function on a subject-by-subject basis and then tested the difference of the log ratios from zero using a one-sample *t* test with FDR correction, as reported previously in a metagenome versus metatranscriptome comparison [7]. On the whole, the percentage of differential features out of the total was extremely high when considering functions (Additional file 1: Table S4), confirming the divergence between potential and active GM functional traits.

**Fig. 3:**
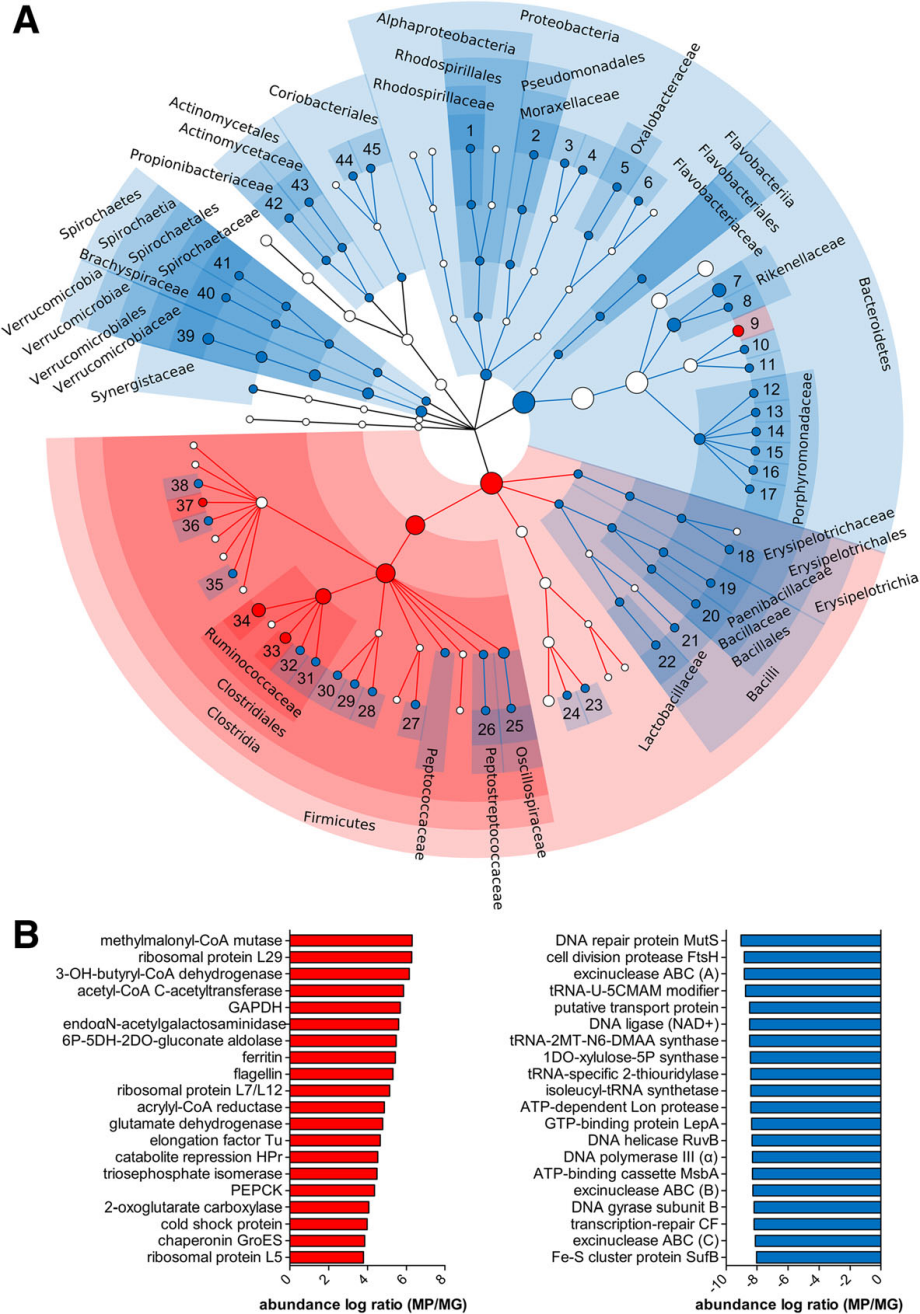
Features with significantly differential abundance between gut metaproteome and metagenome. Data were filtered based on the mean relative abundance of each feature in the sample cohort (threshold >0.01%). **a** Cladogram illustrating differentially abundant taxa (*blue* more abundant in MG, *red* more abundant in MP). Dot size is proportional to the mean relative abundance of the corresponding taxon. *1 Azospirillum*, *2 Acinetobacter*, *3 Escherichia*, *4 Enterobacter*, *5 Oxalobacter*, *6 Parasutterella*, *7 Alistipes*, *8 Mucinivorans*, *9 Prevotella*, *10 Alloprevotella*, *11 Paraprevotella*, *12 Porphyromonas*, *13 Barnesiella*, *14 Odoribacter*, *15 Tannerella*, *16 Parabacteroides*, *17 Butyricimonas*, *18 Holdemania*, *19 Paenibacillus*, *20 Bacillus*, *21 Streptococcus*, *22 Lactobacillus*, *23 Megasphaera*, *24 Veillonella*, *25 Oscillibacter*, *26 Peptoclostridium*, *27 Butyricicoccus*, *28 Pseudoflavonifractor*, *29 Intestinimonas*, *30 Flavonifractor*, *31 Ruminiclostridium*, *32 Anaerotruncus*, *33 Ruminococcus*, *34 Faecalibacterium*, *35 Lachnoclostridium*, *36 Butyrivibrio*, *37 Coprococcus*, *38 Tyzzerella*, *39 Akkermansia*, *40 Brachyspira*, *41 Treponema*, *42 Propionibacterium*, *43 Actinomyces*, *44 Eggerthella*, *45 Gordonibacter*. **b** Bar graphs showing the KEGG orthology functional groups with higher MP/MG log ratio (top 20, *left*) and those with lower MP/MG log ratio (top 20, *right*). Subunit names are shown into brackets. *OH* hydroxy, *GAPDH* glyceraldehyde 3-phosphate dehydrogenase, *6P-5DH-2DO-gluconate* 6-phospho-5-dehydro-2-deoxy-d-gluconate, *PEPCK* phosphoenolpyruvate carboxykinase, *U-5CMAM modifier* uridine 5-carboxymethylaminomethyl modification enzyme, *2MT-N6-DMAA* 2-methylthio-*N*6-dimethylallyladenosine, *1DO-xylulose-5P* 1-deoxy-d-xylulose-5-phosphate, *CF* coupling factor

Focusing on taxonomy (Fig. 3a), many key GM taxa showed significant differences in relative abundance when comparing gene potential and expressed proteins across the cohort. For instance, Proteobacteria, Spirochaetes, Verrucomicrobia, and Coriobacteriales showed a significantly low log MP/MG ratio, whereas taxa belonging to Firmicutes and Bacteroidetes behaved more heterogeneously. Among them, *Faecalibacterium* and *Ruminococcus* (Firmicutes) as well as *Prevotella* (Bacteroidetes) exhibited a significantly high log MP/MG ratio, while a significantly low log MP/MG ratio was measured for Bacilli and Erysipelotrichia (Firmicutes), as well as for Rikenellaceae and Porphyromonadaceae (Bacteroidetes).

The top differential functions are illustrated in Fig. 3b (see Additional file 3: Dataset S1 for further details). Several enzymatic functions exerted by the GM of the studied cohort presented a significantly high log MP/MG ratio, as those involved in short-chain fatty acid (SCFA, including propionate and, mostly, butyrate) metabolism, as well as in degradation of carbohydrates, polyols, and organic acids. Mapping differential KOGs in the carbon metabolism KEGG map (Additional file 4: Figure S2) visually illustrates that the most active metabolic activities performed by the GM are related to glycolysis, gluconeogenesis, pentose phosphate pathway, and butyrate biosynthesis. Ferritin and flagellin were the non-enzymatic proteins with the highest MP/MG log ratio. On the other hand, functions with the lowest MP/MG log ratios were related to amino acid, transfer RNA (tRNA), and cell wall biosynthesis, as well as to DNA replication and repair. Since some of the MG sequences matching with a high number of peptides did not present any KOG functional annotation, we performed an additional differential analysis simply considering the gene/protein name as functional information. As a result, two previously non-annotated proteins (propanediol utilization protein PduA and reverse rubrerythrin) exhibited significantly (and extremely) high MP/MG log ratios, indicating their massive expression yield within the subjects’ GMs.

### Conserved and variable features in the gut microbiota

Another indication provided by Fig. 2 was that MG data generally exhibited a much higher inter-individual variability (expressed by box width) in taxa than in potential functions, whereas this trend could not be observed in MP. To quantify this observation in a more rigorous and comprehensive fashion, we computed between-subject dissimilarity (Bray-Curtis index) at the taxonomic (genus) and functional level for both MG and MP data. As a confirmation, a higher inter-individual variability concerning the taxonomic composition could be measured in MG compared to MP (Wilcoxon signed-rank test with continuity correction, two-tailed *P* = 4.7 × 10^−8^), while the analysis of functional data revealed a higher variability in MP than in MG (*P* < 2.2 × 10^−16^).

To further assess which specific GM taxa and functions were more conserved and variable within the human cohort under study, we also calculated the abundance coefficient of variation (CV) for each taxon and function measured by MG and/or MP across the 15 subjects. We set two arbitrary thresholds (CV > 150% and < 60%) to define features with high and low inter-individual variability, respectively. The amount of high and low variability features was similar between MG and MP (about 30 and 10%, respectively); conversely, and consistently with dissimilarity data, GM expressed functions (MP) were globally much more variable in abundance within the population compared to the potential functions (MG), even though this effect was less evident when weighing each feature based on its abundance (Additional file 1: Table S5).

**Fig. 4:**
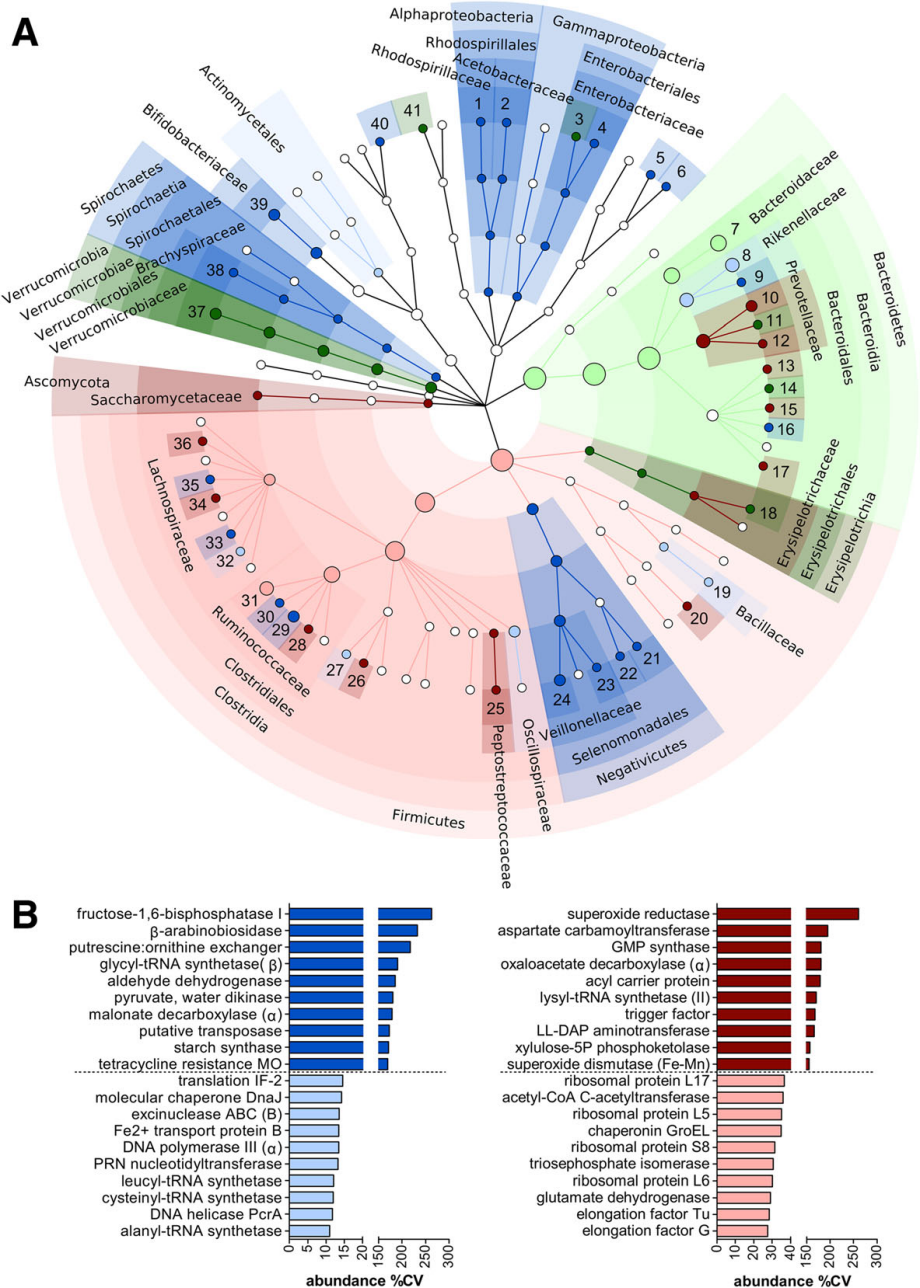
Inter-individual variability of gut microbiota features. Data were filtered based on the mean relative abundance of the features in the sample cohort (threshold >0.01%). **a** Cladogram illustrating taxa with CV >150% (variable, *darker color*) or <60% (conserved, *lighter color*) across subjects, according to MG (*blue*) and MP (*red*) data. Green dots represent taxa found conserved (*dark*) or variable (*light*) based on both MG and MP data. Dot size is proportional to the mean relative abundance of the corresponding taxon. *1 Azospirillum*, *2 Acetobacter*, *3 Escherichia*, *4 Enterobacter*, *5 Parasutterella*, *6 Sutterella*, *7 Bacteroides*, *8 Alistipes*, *9 Mucinivorans*, *10 Prevotella*, *11 Alloprevotella*, *12 Paraprevotella*, *13 Porphyromonas*, *14 Barnesiella*, *15 Odoribacter*, *16 Tannerella*, *17 Butyricimonas*, *18 Holdemanella*, *19 Bacillus*, *20 Streptococcus*, *21 Acidaminococcus*, *22 Phascolarctobacterium*, *23 Megasphaera*, *24 Dialister*, *25 Peptoclostridium*, *26 Intestinimonas*, *27 Flavonifractor*, *28 Anaerotruncus*, *29 Ruminococcus*, *30 Subdoligranulum*, *31 Faecalibacterium*, *32 Lachnoclostridium*, *33 Dorea*, *34 Butyrivibrio*, *35 Coprococcus*, *36 Marvinbryantia*, *37 Akkermansia*, *38 Brachyspira*, *39 Bifidobacterium*, *40 Gordonibacter*, *41 Desulfovibrio*. **b** Bar graphs showing the 10 KEGG orthology functional groups with higher CV (variable, *darker color*) and the 10 with lower CV (conserved, *lighter color*) across subjects, according to MG (*left*, *blue*) and MP (*right*, *red*) data. Subunit names are shown into brackets. Only functional groups detected in at least half of the subjects are shown. *MO* monooxygenase, *IF* initiation factor, *PRN* polyribonucleotide, *GMP* guanosine monophosphate, *LL-DAP* l,l-diaminopimelate, *5P* 5-phosphate

Focusing on taxonomy (cladogram in Fig. 4a), a moderate correlation could be observed between MG and MP concerning taxa abundance variability (*ρ* = 0.33), although no taxa showed opposite trends (e.g., low variability with MG and high variability with MP). MG and MP provided consistent results for the taxonomic lineage from Verrucomicrobia to *Akkermansia*, which exhibited high variability within the subjects, and for the taxonomic lineage from Bacteroidetes to *Bacteroides*, which was instead rather conserved. Moreover, although with slight differences in the degree of variability between MG and MP, levels of *Bifidobacterium*, *Prevotella*, and *Butyrivibrio* displayed a considerably high variability across the cohort under study, while *Alistipes* and *Faecalibacterium* were found to be rather conserved in abundance among the subjects analyzed.

The most conserved and variable functions (KOGs) are shown in Fig. 4b. A very weak correlation could be observed between MG and MP concerning function abundance variability (*ρ* = 0.12). In general, the abundance of genes related to tRNA and peptidoglycan synthesis, as well as to DNA replication and repair, showed low variability among subjects, contrary to some potential activities (including transposases, antibiotic resistance genes, and enzymes involved in catabolism of glycans and biogenic amines) exhibiting a higher variability. On the other hand, functions related to glutamate degradation and biosynthesis of butyrate, besides “housekeeping” glycolytic enzymes and translation factors, appeared to be consistently active in all subjects (with high abundance and low variability) based on MP data; interestingly, several stress-related proteins (such as superoxide scavengers and a trigger factor) were found to be among the most variable GM features within the population. Complete data can be found in Additional file 3: Dataset S1.

### Specific functional contribution of Firmicutes and Bacteroidetes

**Fig. 5:**
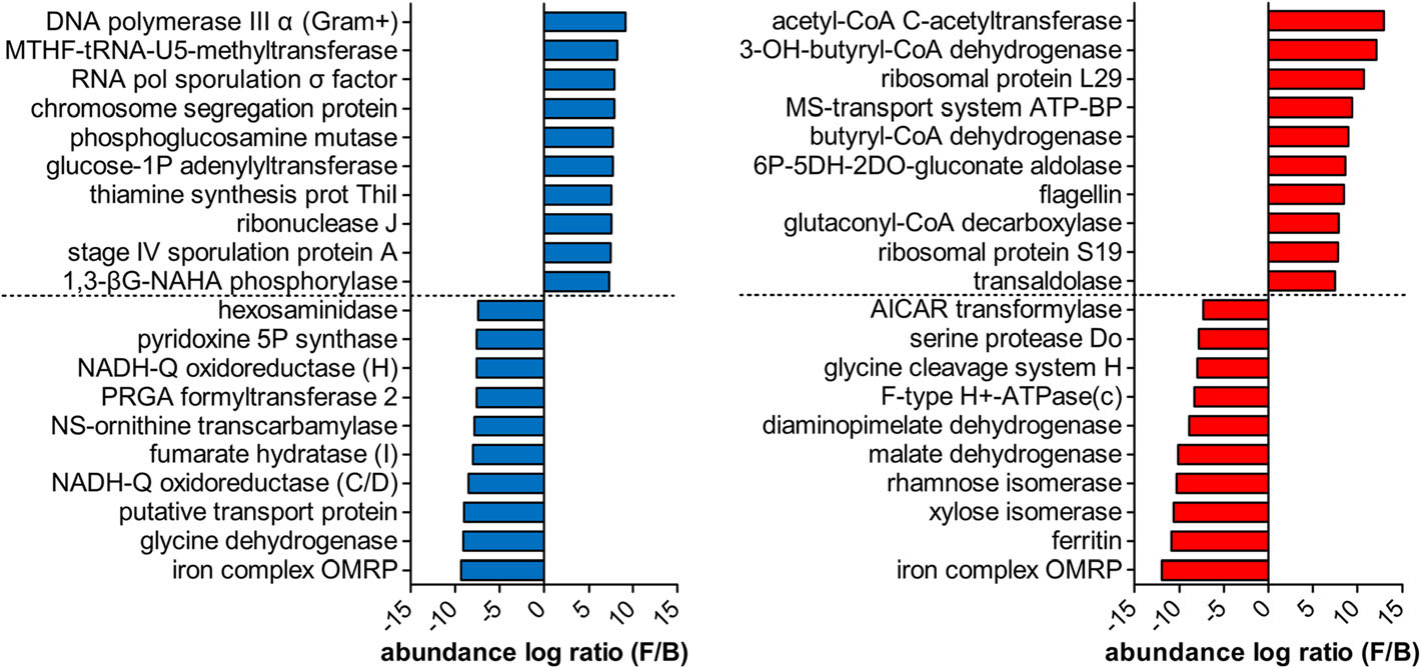
KEGG orthology functional groups with significantly differential abundance between Firmicutes and Bacteroidetes. Data were filtered based on the mean relative abundance of the features in the sample cohort (threshold >0.01%). Functions with higher (top 10) and lower (top 10) Firmicutes/Bacteroidetes (F/B) log ratio according to MG data are shown in the *left* bar graph (*blue*); functions with higher (top 10) and lower (top 10) F/B log ratio according to MP data are shown in the *right* bar graph (*red*). Subunit names are shown into brackets. *MTHF-tRNA-U5* methylenetetrahydrofolate-tRNA-(uracil-5), *1P* 1-phosphate, *1,3-β G-NAHA* 1,3-beta-galactosyl-*N*-acetylhexosamine, *5P* 5-phosphate, *NADH-Q* NADH-quinone, *PRGA* phosphoribosylglycinamide, *NS-ornithine N*-succinyl-l-ornithine, *OMRP* outermembrane receptor protein, *OH* hydroxy, *MS-transport* multiple sugar transport, *ATP-BP* adenosine triphosphate-binding protein, *6P-5DH-2DO-gluconate* 6-phospho-5-dehydro-2-deoxy-d-gluconate, *AICAR* 5-aminoimidazole-4-carboxamide ribonucleotide

We also sought to find phylum-specific functions, i.e., activities mainly or exclusively due to one of the main GM phyla (Firmicutes and Bacteroidetes). To this aim, we computed the log Firmicutes/Bacteroidetes (F/B) abundance ratio for each function on a subject-by-subject basis, as described above for the MG versus MP comparison. Bar graphs of Fig. 5 illustrate functions with the highest and lowest log F/B ratios, according to MG (left, blue) and MP (right, red) data (the complete lists of differential features are given in Additional file 5: Dataset S2). Phylum-specific genes within the metagenome, providing insights into the peculiar functional potential of Firmicutes and Bacteroidetes members across the cohort under study, belonged to a wealth of different activities (including sporulation, cell wall biogenesis and ion transport), mapping to several relevant biosynthetic and degradative pathways (the related carbon metabolism pathway map is reported in Additional file 6: Figure S3). When considering the metaproteome, the specific contribution of the two main GM phyla appears to be better defined and oriented towards more interrelated metabolic activities. While Bacteroidetes were found to be specifically involved in multiple activities, including iron homeostasis, catabolism of non-glucose monosaccharides (rhamnose, xylose), and folate metabolism, Firmicutes’ specific contribution to the GM metabolism was mainly in butyrate biosynthesis, being most of the differential enzymes (including acetyl-CoA C-acetyltransferase, 3-hydroxybutyryl-CoA dehydrogenase, butyryl-CoA dehydrogenase, glutaconyl-CoA decarboxylase, and enoyl-CoA hydratase) eventually converging on butyrate production (as illustrated also in the carbon metabolism KEGG map of Additional file 7: Figure S4).

### Active role of main gut microbiota members in the carbohydrate metabolism

**Fig. 6:**
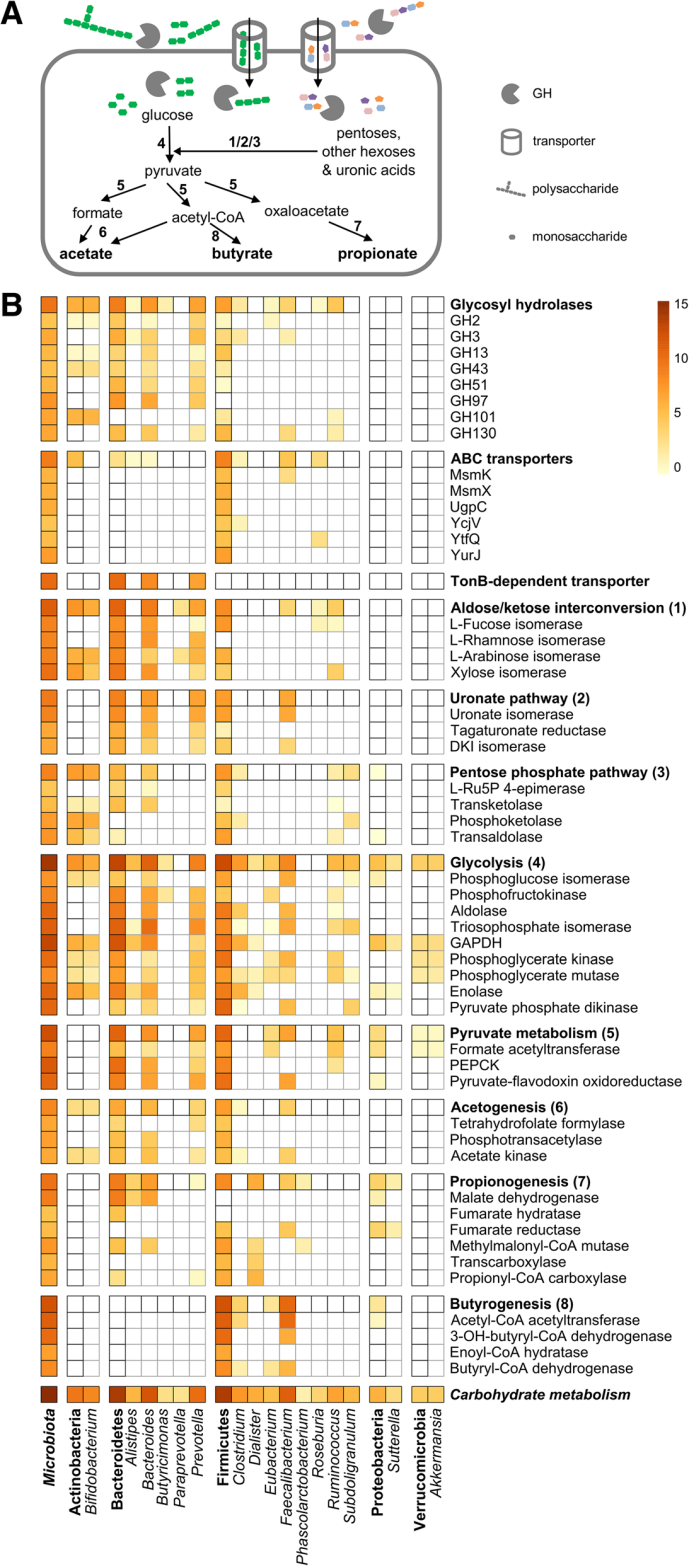
Active carbohydrate metabolism pathways and related taxonomic assignments. **a** Schematic overview of gut microbiota metabolic pathways from carbohydrate uptake and degradation to the production of short-chain fatty acids (in *bold*). *Numbers in bold* correspond to the metabolic pathways listed in **b**. *GH* glycosyl hydrolase. **b** Combination of carbohydrate metabolism pathways/enzymes (*rows*) and specific gut microbiota phyla/genera (*columns*) found by MP analysis. Heatmap color scale is based on the logarithmized relative abundance (average of 15 subjects) of each function-taxon combination. For each pathway (*rows*), only enzymes detected in at least half of the subjects are reported, while the *top row* (in *bold*, corresponding to *black-bordered squares*) accounts for the total abundance of all enzymes (found in at least one subject) belonging to the pathway. For each phylum (*columns*), only genera expressing a function in at least two subjects are reported, and the phylum column (in *bold*, corresponding to *black-bordered squares*) accounts for the total abundance of all functions assigned to that given phylum. “Carbohydrate metabolism” and “microbiota” report the total of rows and columns, respectively. *GH* glycosyl hydrolase, *ABC* ATP-binding cassette, *MsmK* multiple sugar-binding transport ATP-binding protein MsmK, *MsmX* maltodextrin import ATP-binding protein MsmX, *UgpC* sn-glycerol-3-phosphate import ATP-binding protein UgpC, *YcjV* uncharacterized ABC transporter ATP-binding protein YcjV, *YtfQ* ABC transporter periplasmic-binding protein YtfQ, *YurJ* uncharacterized ABC transporter ATP-binding protein YurJ, *DKI* 4-deoxy-l-threo-5-hexosulose-uronate ketol, *Ru5P* ribose-5-phosphate, *GAPDH* glyceraldehyde 3-phosphate dehydrogenase, *PEPCK* phosphoenolpyruvate carboxykinase, *OH* hydroxy

To further elucidate the specific role of the main GM members within carbohydrate metabolism, we manually parsed functional and taxonomic annotations of transporters and enzymes identified by MP and responsible for processes ranging from polysaccharide degradation to SCFA production. As schematized in Fig. 6a, complex polysaccharides are usually degraded in the extracellular space, then oligo- and monosaccharides are transported inside the microbial cell, where they are degraded through carbohydrate catabolic pathways (converging on glycolysis); pyruvate and related intermediates are finally utilized for the biosynthesis of SCFAs, including acetate, propionate, and butyrate. Figure 6b illustrates the expression level of each function-taxonomy combination, with functions grouped according to the reference pathway (or functional family), and microbial genera grouped according to the corresponding phylum. Overall pathway results were retrieved from MP expression data of 81 functional groups (KOGs); 51 of them, found to be expressed in at least half of the subjects, are also reported as single functions.

Considering the GM as a whole, the expression of glycolytic enzymes accounted for about half of the total carbohydrate metabolism, while the relative contribution of butyrate, propionate, and acetate biosynthesis enzymes was 12, 3, and 1%, respectively. Another relevant metabolic activity was aldose/ketose interconversion (7%), whereas sugar transporters (comprising TonB-dependent transporters from Bacteroidetes and ABC transporters from Firmicutes) accounted in total for 6% of carbohydrate metabolism-related proteins. Carbohydrate metabolism appeared to be due to Firmicutes and Bacteroidetes at similar extents (46 and 51% of the total, respectively), with Actinobacteria playing a minor role in quantitative terms (3%).

When focusing on the metabolic tasks performed by specific GM members, *Bifidobacterium* was found to contribute significantly to mucin glycoprotein degradation (endo-α-*N*-acetylgalactosaminidase activity), as well as to pentose hydrolysis (beta-xylosidase), interconversion (pentose isomerases), and catabolism (phosphoketolase and transaldolase within the pentose phosphate pathway). *Bacteroides* spp. provided a peculiar and active contribution to starch degradation and uptake, mainly through enzymes and transporters belonging to the starch utilization system (Sus), and were clearly shown to play a key role in fucose, rhamnose, and uronate metabolism and also in the glycolytic pathway (especially in the preparatory phase); another main member of Bacteroidetes, *Prevotella*, was instead primarily involved in xyloglucan and arabinan degradation. Among Firmicutes genera, we found a strong (and almost exclusive) involvement of *Faecalibacterium* in butyrogenesis (as well as in oligosaccharide membrane transport and pyruvate phosphate dikinase activity) and of *Dialister* in the final part of propionogenesis; furthermore, a considerably high formate C-acetyltransferase activity from *Ruminococcus* spp. was observed. Of note, a high level of sequence homology was observed for many orthologous genes within the same phylum (especially pentose phosphate pathway enzymes and ABC transporters expressed by Firmicutes spp.), making it difficult to achieve a taxonomic classification down to the genus level (at least through the lowest common ancestor approach employed here).

## Discussion

This study was meant as a comparative and systematic investigation of potentially and actually expressed features in the GM of a healthy human population. To this purpose, a cohort of clinically monitored Sardinian subjects, following an omnivorous diet and with a BMI distribution largely comparable to that of the general Italian population, was selected for stool sample collection and GM characterization through shotgun MG and MP. As both diet and BMI are known to deeply influence the GM composition (as well as, most likely, its activity), we cannot rule out that cohorts with different food regimens and/or BMI distributions may show different taxonomic and functional profiles; however, a specific investigation of these aspects falls out of the scope of the present work. Furthermore, gut microbiota characterization was carried out using fecal samples, as stool can be collected following non-invasive procedures and is widely recognized as a good proxy for the colonic microbial mass. Nevertheless, some differences in structure and functional expression between colonic and fecal microbial communities are expected, especially concerning oxygen-sensitive species and enzymes [10, 11].

The taxonomic composition of the GM of the studied cohort, based on MG and MP data, exhibited a large inter-individual variability at the phylum level, with Firmicutes ranging from 6 to 78% and Bacteroidetes ranging from 21 to 88% (and the F/B ratio ranging from 0.06 to 3.63), as clearly described in previous studies [2, 12, 13]. The GM taxonomic profile is known to vary widely among different cohorts, based on both genetic and environmental features, including dietary and cultural habits [14–17]. Moreover, it is worth reminding that the output of meta-omic taxonomic profiling can be largely influenced by the DNA/protein extraction methods, by library preparation methodologies, and by the specific sequence database(s) used for taxonomic annotation (as detailed below) [18–21].

Another interesting indication provided by this work, although obtained in a small population, regards the inter-individual variability of the abundance of specific GM members. In particular, the relative level of several key genera, including *Akkermansia*, *Prevotella*, and *Bifidobacterium*, was globally poorly conserved within the human cohort studied, according to both MG and MP results, suggesting a possible higher responsiveness to variables like diet or other environmental factors. Consistently, *Akkermansia* abundance has been recently observed to be significantly modulated by many different foods and dietary variables [22–25], changes in *Prevotella* spp. levels have been related to increase in fibers in the diet and to glucose metabolism and tolerance [26–28], and many bifidobacteria are widely and long used as probiotics due to their (purported) ability to induce/restore GM homeostasis [29, 30]. Of note is also the finding concerning the low and high inter-individual variability of butyrate biosynthesis enzymes (see below for discussion on the role of butyrate in gut health) and oxidative stress-related GM functions, respectively. Response of microorganisms to reactive oxygen species may in fact vary among individuals based on several factors, including the degree of activation of oxidative stress mechanisms modulated by the host immune system [31, 32]. Moreover, we found that *Faecalibacterium*, *Ruminococcus*, and *Prevotella* exhibited a high log MP/MG ratio, suggesting a high protein expression activity of these taxa. This was previously observed for *Faecalibacterium*, when comparing abundance data based on 16S rRNA gene analysis with MP results [33].

A considerable divergence between GM functional potential and activity was also observed in the present study. Multiple data analysis approaches consistently revealed that the GM protein expression pattern differs significantly from that of the gene potential, both in terms of feature abundance distribution and inter-individual variability. Since the very beginning of GM research, there was evidence for similarity among individuals in MG functional profiles and metabolic pathway gene modules, despite variation in community structure [2], while, in a pioneering study, VerBerkmoes and coworkers described a more uneven distribution of functional categories in the human stool metaproteome compared to a (non-matched) metagenome [5]. Similar conclusions were drawn by Franzosa et al. when comparing human gut metatranscriptome and metagenome [7], even if the correlation between MT and MG functional datasets was much higher than that measured between MP and MG in this study, in line with recent reports [6]. The higher inter-individual variability in GM protein functions compared to the corresponding gene functions (even considering that the GM taxonomic structure exhibited an opposite behavior) confirms that the metaproteome displays a higher plasticity, being thus a preferential indicator of functional changes in the GM when compared to MG approaches. Concerning this divergence between MG and MP data, it is worth noting that some possible variables might influence the consensus between the two omic approaches. Among them, the possible impact of differences in genome size among microbial species should not be overlooked [34], as well as the influence of potentially varying redundancy levels among functionally relevant genes [35]. The different information depths which can be reached by MP and MG (at least with the currently available technological and bioinformatic approaches) should also be considered as a factor that might lead to a lower quantitative correlation between the two datasets [6]. As a further consideration, it is worth observing that MG keeps the ability of providing the full functional history of bacteria travelling the gut, whereas MP allows the investigator to take a picture of the GM activity at a given time point, location, or condition; this undoubtedly makes these two approaches complementary when attempting to fully characterize the GM functionality.

When comparing the most abundant protein functions observed in this study with earlier MP investigations of healthy human GMs [33, 36], a general consistency could be found. In particular, glutamate dehydrogenase was the most abundant protein function revealed in this study, as previously reported by Kolmeder and coworkers [33, 37]. Bacterial glutamate dehydrogenase plays a pivotal role in the intermediary metabolism in bacteria as well as in animals, providing a major biosynthetic pathway for glutamate production. Glutamate, in turn, is key as a link between carbon and nitrogen metabolism and has been recently shown to be important for *Clostridium difficile* colonization of human gut [38]. Hence, given the abundance of glutamate dehydrogenase, its impact might be relevant for colonization and survival of many other taxa that inhabit the human intestine. Finally, the glutamate circuit has been proposed as central to the neuro-endocrinological role of gut microbiota, the signaling to the CNS through the intestinal epithelial cell glutamate receptors, and the activation of the vagal route [39]. Other abundant functions, which were included among the top functional categories in previous studies [5, 33], are glyceraldehyde 3-phosphate dehydrogenase, phosphoenolpyruvate carboxykinase, acetyl-CoA C-acetyltransferase, enolase, and many other enzymes responsible for essential steps of glycolysis and butyrogenesis, supporting the hypothesis that these functions and pathways are key for the intestinal homeostasis. In particular, the massive and stable expression of butyrate biosynthesis enzymes by Firmicutes (mainly *Faecalibacterium*) deserves key attention. Butyrate is a four-carbon SCFA known as one of the main products of microbial fermentation in the human colon and serves as preferential energy source for colonocytes [40]. Growing experimental evidences support the importance of butyrate for colon health, for instance highlighting the relationship between defects in butyrate production and pathogenesis or severity of inflammatory bowel diseases and obesity-related metabolic diseases [41–43]. Our results demonstrate a high and constant butyrogenetic activity within a healthy human cohort, in line with previous reports showing a higher abundance of butyrate biosynthetic enzymes compared to those involved in propionate biosynthesis in a healthy cohort [33], thus further supporting GM butyrate production as a key requirement for intestinal health. Further, we observed an apparent pivotal role of *Faecalibacterium* spp. in synthesizing the enzymes involved in this metabolic pathway. It needs to be noted, however, that enzyme abundance may not be directly correlated to metabolite concentrations, as metabolic fluxes are strongly influenced by enzyme kinetics and substrate availabilities [44]. Future metametabolomic investigations are therefore expected to shed further light on these key aspects and their impact on GM metabolism.

In addition, elongation factor (EF)-Tu and chaperonin GroES were the second and the fifth most abundant functions observed in this study. EF-Tu is known for a long time to be among the most abundant proteins in many bacterial species, and, therefore, its highest abundance was not unexpected. GroES, together with GroEL, is also known to be abundantly present in bacteria, where it serves for proper folding of many proteins, particularly when large loads of deleterious mutations occur. Bacterial chaperonin overexpression is hypothesized to be required when bacterial communities evolve under a strong genetic drift [45]. According to the ecological model proposed by Schloissnig and colleagues, this is the case of the human gut microbiome, where a large selection of bacterial population members is subjected to community shifts during the different stage of the individual host life, and, therefore, their extinction might be rescued in the presence of abundant chaperonins [46].

Carbohydrate metabolism was proved in earlier works as the most represented COG category in the human gut metaproteome, highlighting its functional relevance in the host-microbiota interplay [5, 33]. In this respect, the thorough reconstruction of the GM carbohydrate metabolism pathways presented here, including the active contribution to it of the main GM taxa, provides useful insights into the catabolism and cross-feeding networks actually occurring in a healthy human GM. There is evidence that both generalist (able to degrade a wide range of carbohydrates) and specialist (able to target only a few selected glycans) members of the GM belong to metabolic networks where cross-feeding takes place, since by-products of one microorganism can serve as key metabolic resources for other GM members [47]. The activity of sugar-converting enzymes deserves special attention in this respect. l-fucose isomerase was described as expressed at high levels in the GM of healthy individuals in two earlier studies [5, 33]; furthermore, both l-arabinose and uronate metabolism were found to be particularly active in one of these previously analyzed human cohorts [33]. Here, we were also able to assign these metabolic functions to the main microbial taxa actively expressing them, showing a general active involvement of *Faecalibacterium*, *Bacteroides*, and *Prevotella* (at different extents) both in the aldose/ketose interconversion and in the uronate pathway, and peculiar (and possibly interrelated) roles of other genera in specific enzymatic activities (e.g., isomerization of l-arabinose and xylose for *Bifidobacterium*, l-fucose and xylose for *Ruminococcus*). It is also worth noting that enzymes involved in methanogenesis and sulfate reduction were detected at much lower abundance when compared to GMs from other human cohorts [7], or even from different hosts, such as murine models [19], highlighting the impact of genetics, diet, and gut anatomy on the GM structure and metabolic functions, which can be captured and quantified by means of (multi-)meta-omic approaches.

The choice of a proper sequence database is a key issue in MP, as it might have a strong influence both on identification and annotation yields [48–50]. Recently, we observed that the use of experimental MG sequences as MP databases can be useful when dealing with human samples, and that employing population-based databases (i.e., combining all MG sequences from the population under study) provides better results than sample-matched databases [21]. In keeping with these indications, a population-based database, containing all MG read sequences retrieved from the same cohort under study, was used in this work, with the aim of measuring the protein expression rate of a given metagenome. We decided not to apply any sequence assembly strategy to the reads, although this has been found as beneficial for functional annotation efficiency and would have led to a slight increase in peptide identifications (data not shown), as it would have dramatically reduced the dynamic range of MG counts, unless using really complex and computationally demanding co-assembly strategies [6]. Concerning functional annotation, we adopted the same strategy recently applied on human and mouse GM datasets upon critical evaluations [21]. We acknowledge that the global functional annotation yield of MG reads presented here is rather low, although the presence of a considerable amount of unknown and poorly annotated functions is a known issue in metaproteomics. Concerning taxonomic classification, the global annotation yield was satisfactory, although a non-negligible portion of functional families, having a high level of sequence homology within related taxa, could only be mapped to a specific phylum, but not to lower levels (e.g., genus). This appears to be likely due to intrinsic limitations of both shotgun mass spectrometry (being the length of tryptic peptides quite limited) and the lowest common ancestor approach used for annotation (which is not able to discriminate among closely related orthologs). Improved taxonomic and functional annotation yields might be expected using different sequencing platforms allowing for the generation of longer reads, as well as employing novel and better performing annotation tools and databases which are hopefully going to be released in the near future. Finally, we cannot also ignore a possible impact of technical variability on the results (although Illumina sequencing protocols, as well as the shotgun MP pipeline used [19], are established and fairly reproducible), and the use of read and spectral counts as quantitative measure of abundance should be recognized as an estimation, although quite robust and widely used [51, 52], rather than an actual quantification.

## Conclusions

We found that a considerable divergence exists between functional potential and expression in the GM of a healthy human cohort. Furthermore, our results give insights into the understanding of active functions and metabolic tasks of a “normal” GM, highlighting the overall key importance of butyrate production. A detailed picture is also provided about the specific contribution of GM taxa to the main functional activities, focusing on carbohydrate metabolism. Our data suggest that caution should be used before drawing conclusions on the actual GM functional activity based on metagenomic data, and support MP as a valuable approach to investigate the functional role of the GM in health and disease.

## Methods

### Samples

Stool samples were collected from 15 healthy Sardinian volunteers (8 females and 7 males) from the SardiNIA cohort population. Briefly, the SardiNIA study investigates genotypic and phenotypic aging-related traits in a longitudinal manner. All residents from 4 towns (Lanusei, Arzana, Ilbono, and Elini) in a valley in Sardinia (Italy) were invited to participate. Since November 2001, a total of 6921 individuals aged 18–102 (>60% of the population eligible for recruitment in the area) were recruited and the majority of them (*n* = 6602) have been assessed for ~13.6 million genetic variants [15]. As detailed in Additional file 1: Table S1, the median age of the studied subjects at the time of sampling was 39 years (range 22–48), while their median body mass index (BMI) value was 23.2 (range 18.4–31.2), with a global distribution widely comparable to that of the general Italian population at the time of sampling (source: ISTAT 2014). All samples were immediately stored at −80 °C, then transferred to the Porto Conte Ricerche laboratories in dry ice, and stored again at −80 °C until use. Then, samples were thawed at 4 °C and, from each of them, two equal stool portions (weighing approximately 250 mg each) were collected, of which the first was subjected to DNA extraction for MG analysis and the second underwent protein extraction for MP analysis.

### DNA sample preparation and metagenome sequencing

DNA extraction was undertaken using the QIAamp DNA Stool Mini Kit (Qiagen, Hilden, Germany), according to the manufacturer’s protocol.

Libraries were constructed according to the Nextera XT kit (average insert size ~700 bps) and sequenced with the HiScanSQ sequencer (both from Illumina, San Diego, CA, USA), using the paired-end method and 93 cycles of sequencing.

### Metagenome bioinformatics

Merging and filtering of paired reads were carried out using tools from the USEARCH suite v.8.1.1861 [53, 54] as described previously [21]. The mean length of the paired-end merged reads was 134 bps. Since sequencing depth may affect estimation of the relative abundances of gene categories, filtered reads were subjected to random subsampling using the fastx_subsample command (sample_size 200000). A subsequent evaluation of the taxonomic and functional information depth revealed that 96% of taxa and 98% of KEGG functions with relative abundance >0.01% in the non-subsampled dataset were maintained upon subsampling. Taxonomic annotation was performed using MEGAN v.5.11.3 [55]. Read sequences were preliminary subjected to DIAMOND (v.0.7.1) search against the NCBI-nr DB (2016/03 update), using the blastx command with default parameters [56]. Then, DIAMOND results were parsed using MEGAN to perform lowest common ancestor classification according to default parameters.

Functional annotation was carried out through a DIAMOND blastx search (top hit and e-value threshold 10^−5^) against bacterial sequences from the UniProt/Swiss-Prot database (release 2015_12), followed by retrieval of KEGG orthologous group information associated with each UniProt/Swiss-Prot accession number [57].

The relative abundance of a taxon/function in a subject was calculated by summing the number of reads assigned to that taxon/function and then by dividing the taxon/function read count by the total read count of the subject. Only taxa and functions with a relative abundance higher than 0.01% were considered for subsequent differential analysis.

### Protein sample preparation and mass spectrometry analysis

Samples were resuspended by vortexing in an SDS-based extraction buffer, heated, and then subjected to a combination of bead-beating and freeze-thawing steps, as illustrated elsewhere [19]. Protein extracts were reduced, alkylated, and digested on-filter according to the filter-aided sample preparation (FASP) protocol [58], with slight modifications reported earlier [59].

An LTQ-Orbitrap Velos mass spectrometer (Thermo Scientific, San Jose, CA, USA) interfaced with an UltiMate 3000 RSLCnano LC system (Thermo Scientific) was used for LC-MS/MS analysis. Peptide separation by LC was carried out as previously described [19], while the mass spectrometer was set up in a data-dependent MS/MS mode with HCD as fragmentation method, as detailed elsewhere [59].

### Metaproteome bioinformatics

Peptide identification was performed using Proteome Discoverer (version 1.4; Thermo Scientific), with Sequest-HT as search engine and Percolator for peptide validation (FDR <1%). Search parameters were set as described previously [60], while the sequence database was composed of the open reading frames (ORFs) found using FragGeneScan (v.1.19, with the training for Illumina sequencing reads with about 0.5% error rate) [61] starting from the MG reads obtained in this study, upon clustering at 100% using the dedicated USEARCH tool (25,328,860 sequences in total).

All ORFs matched with at least an MS spectrum upon database searching (average length 42 amino acids) were subjected to taxonomic and functional classification, following the same procedure described above for the whole metagenome sequences (“Metagenome bioinformatics” section), except using the DIAMOND blastp command instead of blastx.

The relative abundance of a taxon/function in a subject was calculated by summing the number of MS spectral counts matched to all ORFs assigned to that taxon/function and then by dividing the taxon/function count by the total MS spectral counts for all taxa/functions detected in that subject (so that the sum of the abundances of all taxa/functions detected in each subject is 1). Only taxa and functions with a relative abundance higher than 0.01% were considered for subsequent differential analysis.

### Statistical analysis and graph generation

Bray-Curtis dissimilarity values were computed using the R package “vegan.” The Wilcoxon signed-rank test (R package “stats”) was applied with continuity correction to compare Bray-Curtis dissimilarity values between MG and MP. The extent of differential abundance of each feature between two groups (MG versus MP or Firmicutes versus Bacteroidetes) was calculated for each subject and expressed as a relative abundance log ratio, using a correction factor (CF = 10^−5^) to eliminate discontinuity due to missing values. The global log ratio was intended as the mean of the log ratios calculated for each subject. The sets of log ratios were further tested for significant deviation from zero using the one-sample *t* test, and an FDR correction was performed on the nominal two-tailed *P* values following the Benjamini-Hochberg method (*α* = 0.05), as reported previously [7], using the SGoF+ tool v.3.8 [62].

PCA plots and heatmaps were generated using ClustVis (http://biit.cs.ut.ee/clustvis) [63], boxplots were created using BoxPlotR (http://shiny.chemgrid.org/boxplotr) [64], GraphPad Prism (v.5.03) was employed for bar graph generation, and cladograms were produced using GraPhlAn [65] and edited using Inkscape (https://inkscape.org). KEGG pathway maps [66] were customized by uploading KO numbers through the “user data mapping” function on the KEGG website (http://www.kegg.jp).

## Additional files

Additional file 1: Table S1. Gender, age, and BMI of the human subjects selected for the study. **Table S2.** Metrics of metagenome and metaproteome analysis. **Table S3.** Taxonomic and functional annotation yields. **Table S4.** Percentage of taxa and functions with differential abundance between the human gut metagenomes and metaproteomes analyzed in this study. **Table S5.** Percentage distribution of conserved and variable features within the human gut metagenomes and metaproteomes analyzed in this study. (DOCX 35 kb)

Additional file 2: Figure S1. Principal component analysis plots related to taxonomic and functional features. MG data are in blue, while MP data are in red. Each dot (with different shape) represents a different human subject. (*A*) phyla; (*B*) genera; (*C*) KOGs; (*D*) KOG-phylum combinations. (PNG 2001 kb)

Additional file 3: Dataset S1. Abundance and differential data (MG versus MP) at phylum, class, order, family, genus, KOG, KOG/phylum, and KOG/genus level. (XLSX 3588 kb)

Additional file 4: Figure S2. Metabolic functions with differential abundance between MP and MG datasets mapped in the KEGG carbon metabolism pathway. Red arrows indicate enzymes with significantly higher abundance in the MP dataset, while blue arrows indicate enzymes with significantly higher abundance in the MG dataset. (PNG 76 kb)

Additional file 5: Dataset S2. Relative abundance and differential analysis outputs concerning Firmicutes and Bacteroidetes KOGs, according to MG and MP data. (XLSX 101 kb)

Additional file 6: Figure S3. Metabolic functions with differential abundance between Firmicutes and Bacteroidetes according to the MG dataset, mapped in the KEGG carbon metabolism pathway. Purple arrows indicate genes with significantly higher abundance in Firmicutes, orange arrows indicate genes with significantly higher abundance in Bacteroidetes, and gray arrows indicate genes detected in one or both phyla but with no differential abundance. (PNG 37 kb)

Additional file 7: Figure S4. Metabolic functions with differential abundance between Firmicutes and Bacteroidetes according to the MP dataset, mapped in the KEGG carbon metabolism pathway. Purple arrows indicate proteins with significantly higher abundance in Firmicutes, orange arrows indicate proteins with significantly higher abundance in Bacteroidetes, and gray arrows indicate proteins detected in one or both phyla but with no differential abundance. (PNG 38 kb)

## Abbreviations

BMI: Body mass index
CV: Coefficient of variation
F/B ratio: Firmicutes/Bacteroidetes ratio
FASP: Filter-aided sample preparation
GM: Gut microbiota
KOG: KEGG orthologous group
MG: Metagenomics
MP: Metaproteomics
MT: Metatranscriptomics
PSM: Peptide-spectrum match
SCFA: Short-chain fatty acid
