# Supplementary material for: Potential and active functions in the gut microbiota of a healthy human cohort
Source: Microbiome. 2017 Jul 14;5:79. doi: 10.1186/s40168-017-0293-3 (PMC5513205; doi:10.1186/s40168-017-0293-3)
Supplement: Supplementary file 1 — Gender, age, and BMI of the human subjects selected for the study. Table S2. Metrics of metagenome and metaproteome analysis. Table S3. Taxonomic and functional annotation yields. Table S4. Percentage of taxa and functions with differential abundance between the human gut metagenomes and metaproteomes analyzed in this study. Table S5. Percentage distribution of conserved and variable features within the human gut metagenomes and metaproteomes analyzed in this study. (DOCX 35 kb) [file 40168_2017_293_MOESM1_ESM.docx]

**Additional Tables**

**Additional file 1: Table S1.** **Gender, age and BMI of the human subjects selected for the study.**

| **Sample ID** | **gender** | **age** | **BMI** |
| --- | --- | --- | --- |
| S1 | F | 39 | 26.5 |
| S2 | M | 39 | 20.5 |
| S3 | M | 33 | 26.5 |
| S4 | M | 48 | 29.0 |
| S5 | F | 45 | 27.7 |
| S6 | F | 27 | 20.7 |
| S7 | M | 44 | 27.0 |
| S8 | F | 44 | 19.3 |
| S9 | F | 23 | 23.2 |
| S10 | M | 45 | 24.2 |
| S11 | F | 48 | 22.8 |
| S12 | F | 38 | 18.4 |
| S13 | F | 24 | 20.1 |
| S14 | M | 24 | 31.2 |
| S15 | M | 22 | 22.0 |

**Additional file 2: Table S2.** **Metrics of metagenome and metaproteome analysis.**

|  | **subject** | **S1** | **S2** | **S3** | **S4** | **S5** | **S6** | **S7** | **S8** | **S9** | **S10** | **S11** | **S12** | **S13** | **S14** | **S15** | ***Mean*** | ***Median*** |
| --- | --- | --- | --- | --- | --- | --- | --- | --- | --- | --- | --- | --- | --- | --- | --- | --- | --- | --- |
| **MP** | **MS spectra** | 22,212 | 19,771 | 19,769 | 19,612 | 19,620 | 21,217 | 22,158 | 21,651 | 20,224 | 20,755 | 20,089 | 23,131 | 21,833 | 21,037 | 18,414 | **20,773** | **20,816** |
|  | **total PSMs** | 10,438 | 4,215 | 7,407 | 8,201 | 8,441 | 5,946 | 7,010 | 6,422 | 6,403 | 8,154 | 4,035 | 8,004 | 10,919 | 6,725 | 4,749 | **7,138** | **7,010** |
|  | **total PSMs/MS spectra** | 47% | 21% | 37% | 42% | 43% | 28% | 32% | 30% | 32% | 39% | 20% | 35% | 50% | 32% | 26% | **34%** | **32%** |
|  | **phylum-annotated PSMs** | 8,779 | 3,519 | 6,310 | 6,863 | 6,880 | 4,995 | 5,948 | 5,019 | 5,293 | 6,955 | 3,365 | 6,815 | 8,966 | 5,596 | 4,043 | **5,956** | **5,948** |
|  | **genus-annotated PSMs** | 4,628 | 1,594 | 3,729 | 3,382 | 3,194 | 2,759 | 3,746 | 2,061 | 2,433 | 3,499 | 1,652 | 3,466 | 3,214 | 3,029 | 2,030 | **2,961** | **3,194** |
|  | **function-annotated PSMs** | 4,078 | 2,023 | 2,488 | 3,143 | 3,477 | 1,991 | 2,626 | 2,484 | 2,818 | 3,825 | 1,453 | 3,281 | 5,210 | 2,278 | 1,581 | **2,850** | **2,626** |
|  | **function+phylum-annotated PSMs** | 3,408 | 1,635 | 2,009 | 2,536 | 2,806 | 1,630 | 2,207 | 1,897 | 2,261 | 3,174 | 1,167 | 2,718 | 4,226 | 1,866 | 1,327 | **2,324** | **2,207** |
|  | **function+genus-annotated PSMs** | 1,147 | 522 | 769 | 870 | 803 | 667 | 1,124 | 521 | 691 | 1,078 | 360 | 828 | 1,149 | 776 | 442 | **783** | **776** |
|  | **total phyla detected** | 9 | 7 | 9 | 7 | 7 | 6 | 7 | 6 | 5 | 7 | 6 | 5 | 6 | 7 | 6 | **7** | **7** |
|  | **total genera detected** | 31 | 27 | 26 | 28 | 33 | 30 | 28 | 36 | 26 | 25 | 23 | 27 | 32 | 32 | 29 | **29** | **28** |
|  | **total functions detected** | 274 | 174 | 181 | 209 | 237 | 199 | 251 | 212 | 213 | 231 | 138 | 214 | 259 | 214 | 136 | **209** | **213** |
|  | **total phyla detected >0.01%** | 5 | 6 | 7 | 6 | 6 | 5 | 6 | 6 | 4 | 6 | 5 | 4 | 6 | 5 | 5 | **5** | **6** |
|  | **total genera detected >0.01%** | 23 | 27 | 26 | 28 | 33 | 30 | 27 | 36 | 26 | 25 | 23 | 27 | 21 | 31 | 29 | **27** | **27** |
|  | **total functions detected >0.01%** | 274 | 174 | 181 | 209 | 237 | 199 | 251 | 212 | 213 | 231 | 138 | 214 | 259 | 214 | 136 | **209** | **213** |
| **MG** | **total reads** | 5,066,106 | 1,086,450 | 5,115,613 | 3,356,777 | 2,155,967 | 3,384,821 | 1,744,957 | 703,952 | 413,936 | 213,471 | 1,388,270 | 526,178 | 240,610 | 252,510 | 344,037 | **2,077,370** | **1,237,360** |
|  | **subsampled/total reads** | 4% | 18% | 4% | 6% | 9% | 6% | 11% | 28% | 48% | 94% | 14% | 38% | 83% | 79% | 58% | **33%** | **18%** |
|  | **phylum-annotated reads** | 156,721 | 168,780 | 157,171 | 164,938 | 152,517 | 159,637 | 149,487 | 141,217 | 163,981 | 176,100 | 145,094 | 143,496 | 158,690 | 144,305 | 174,604 | **157,116** | **157,171** |
|  | **genus-annotated reads** | 109,872 | 116,363 | 113,317 | 125,067 | 95,041 | 108,636 | 101,889 | 79,093 | 116,637 | 122,527 | 93,918 | 77,461 | 95,441 | 82,809 | 130,255 | **104,555** | **108,636** |
|  | **function-annotated reads** | 24,963 | 29,024 | 22,884 | 23,067 | 25,360 | 27,528 | 27,765 | 25,531 | 27,835 | 25,818 | 26,830 | 28,848 | 28,236 | 27,148 | 28,475 | **26,621** | **27,148** |
|  | **function+phylum-annotated reads** | 23,357 | 27,140 | 21,743 | 22,240 | 23,000 | 25,460 | 25,111 | 22,507 | 25,906 | 24,825 | 24,152 | 26,023 | 26,053 | 23,979 | 27,175 | **24,578** | **24,825** |
|  | **function+genus-annotated reads** | 12,336 | 14,434 | 12,765 | 13,464 | 11,146 | 13,586 | 13,575 | 8,804 | 12,349 | 15,500 | 10,999 | 9,337 | 12,147 | 9,514 | 16,489 | **12,430** | **12,349** |
|  | **total phyla detected** | 23 | 15 | 21 | 15 | 27 | 21 | 21 | 21 | 20 | 11 | 18 | 23 | 22 | 24 | 13 | **20** | **21** |
|  | **total genera detected** | 275 | 192 | 216 | 177 | 283 | 235 | 246 | 311 | 198 | 134 | 213 | 259 | 249 | 270 | 140 | **227** | **235** |
|  | **total functions detected** | 2,636 | 1,826 | 1,744 | 1,550 | 1,752 | 1,910 | 1,574 | 1,674 | 1,987 | 1,436 | 1,597 | 1,477 | 2,452 | 1,592 | 1,495 | **1,780** | **1,674** |
|  | **total phyla detected >0.01%** | 6 | 5 | 6 | 5 | 7 | 6 | 7 | 8 | 4 | 4 | 5 | 6 | 7 | 10 | 5 | **6** | **6** |
|  | **total genera detected >0.01%** | 59 | 44 | 57 | 40 | 62 | 59 | 55 | 76 | 43 | 37 | 44 | 53 | 61 | 59 | 36 | **52** | **55** |
|  | **total functions detected >0.01%** | 1,482 | 1,165 | 1,107 | 1,010 | 1,137 | 1,172 | 1,077 | 1,132 | 1,097 | 994 | 1,003 | 1,033 | 1,423 | 1,111 | 999 | **1,129** | **1,107** |

**Additional file 3: Table S3.** **Taxonomic and functional annotation yields.**

|  | | **MG** | | **MP** | |
| --- | --- | --- | --- | --- | --- |
|  |  | ***mean*** | ***SD*** | ***mean*** | ***SD*** |
| **Taxonomy** | *% of reads/peptides annotated as Bacteria/Archaea* | 81.31% | 4.92% | 95.37% | 1.10% |
|  | *% of reads/peptides annotated as Bacteria/Archaea and further assigned to a specific phylum* | 96.62% | 1.18% | 87.43% | 1.69% |
|  | *% of reads/peptides annotated as Firmicutes and further assigned to a specific genus* | 50.11% | 6.68% | 40.20% | 7.19% |
|  | *% of reads/peptides annotated as Bacteroidetes and further assigned to a specific genus* | 71.13% | 8.30% | 59.33% | 5.69% |
| **Function** | *% of reads/peptides assigned to a specific KOG* | 13.31% | 0.98% | 39.51% | 5.21% |

**Additional file 5: Table S4.** **Percentage of taxa and functions with differential abundance between the human gut metagenomes and metaproteomes analyzed in this study.** The extent of differential abundance of each feature between two groups (MG *vs* MP) was calculated for each subject and expressed as a relative abundance log ratio. The sets of log ratios were further tested for significant deviation from zero using the one-sample *t* test with Benjamini-Hochberg correction for multiple testing. *nd*, not differential

|  |  | **> in MP** | ***nd*** | **> in MG** |
| --- | --- | --- | --- | --- |
| *taxa* | *MG dataset* | 6% | 35% | 59% |
|  | *MP dataset* | 9% | 55% | 36% |
| *KOG* | *MG dataset* | 6% | 5% | 89% |
|  | *MP dataset* | 26% | 28% | 46% |

**Additional file 8: Table S5.** **Percentage distribution of conserved and variable features within the human gut metagenomes and metaproteomes analyzed in this study.**

|  |  | **number of features** | | |
| --- | --- | --- | --- | --- |
|  |  | **cv<60%** | **60%<cv<150%** | **cv>150%** |
| *taxa* | *MG dataset* | 10% | 56% | 34% |
|  | *MP dataset* | 12% | 57% | 31% |
| *KOG* | *MG dataset* | 59% | 39% | 2% |
|  | *MP dataset* | 19% | 49% | 32% |
