# Supplementary figures and images for: Potential and active functions in the gut microbiota of a healthy human cohort
Source: Microbiome. 2017 Jul 14;5:79. doi: 10.1186/s40168-017-0293-3 (PMC5513205; doi:10.1186/s40168-017-0293-3)

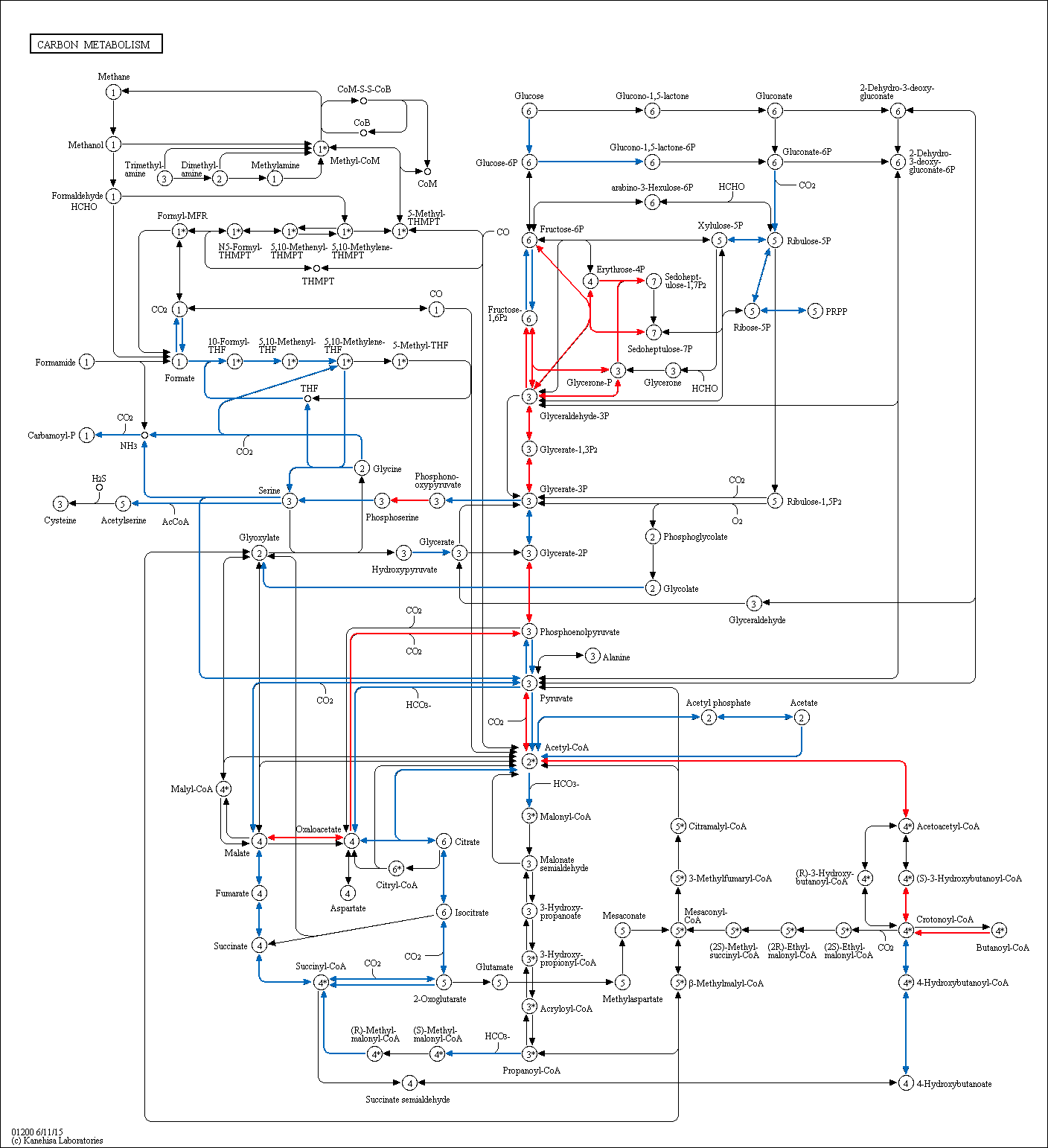

Supplement: Supplementary file 4 — Metabolic functions with differential abundance between MP and MG datasets mapped in the KEGG carbon metabolism pathway. Red arrows indicate enzymes with significantly higher abundance in the MP dataset, while blue arrows indicate enzymes with significantly higher abundance in the MG dataset. (PNG 76 kb) [file 40168_2017_293_MOESM4_ESM.png]

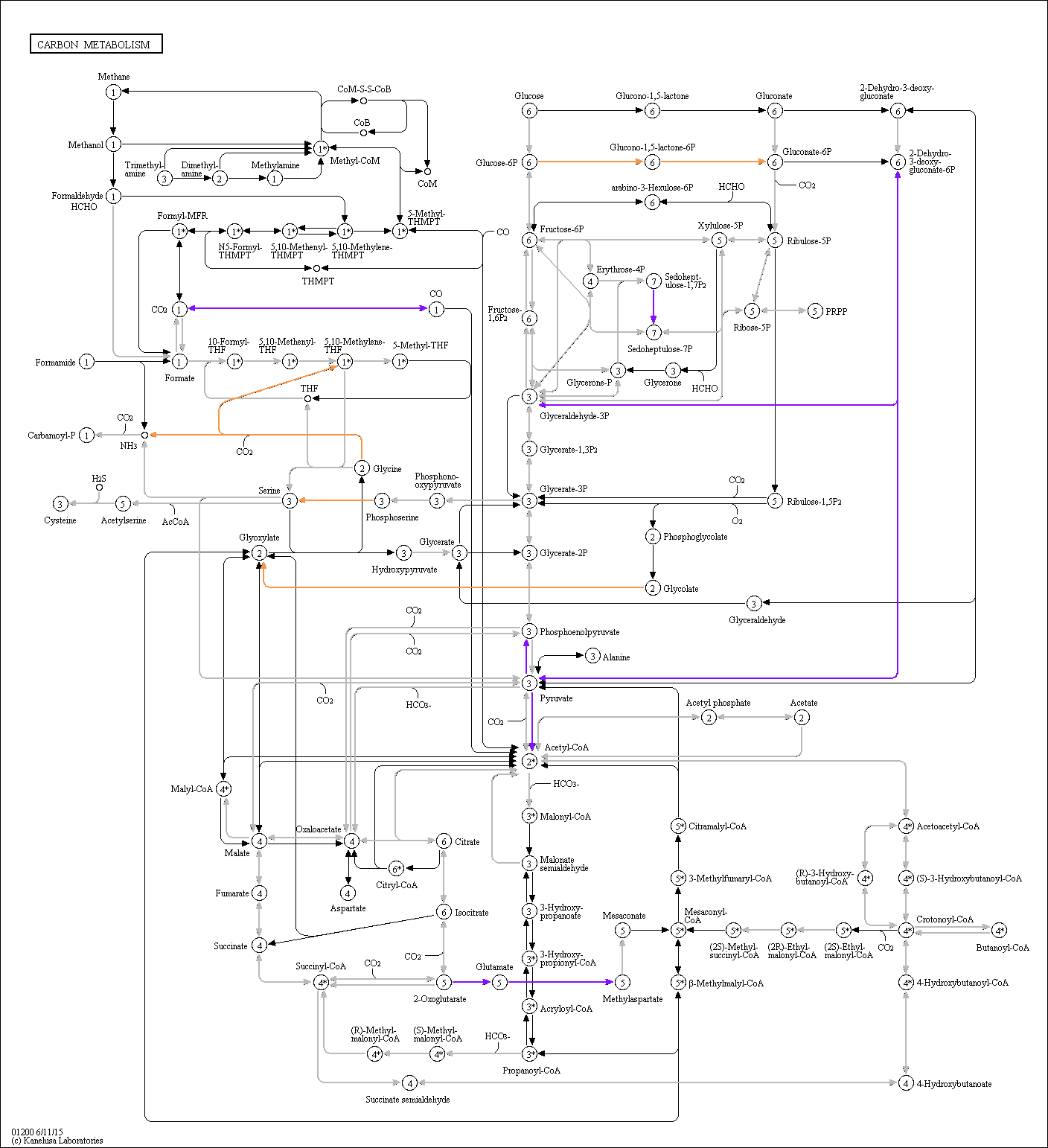

Supplement: Supplementary file 6 — Metabolic functions with differential abundance between Firmicutes and Bacteroidetes according to the MG dataset, mapped in the KEGG carbon metabolism pathway. Purple arrows indicate genes with significantly higher abundance in Firmicutes, orange arrows indicate genes with significantly higher abundance in Bacteroidetes, and gray arrows indicate genes detected in one or both phyla but with no differential abundance. (PNG 37 kb) [file 40168_2017_293_MOESM6_ESM.png]

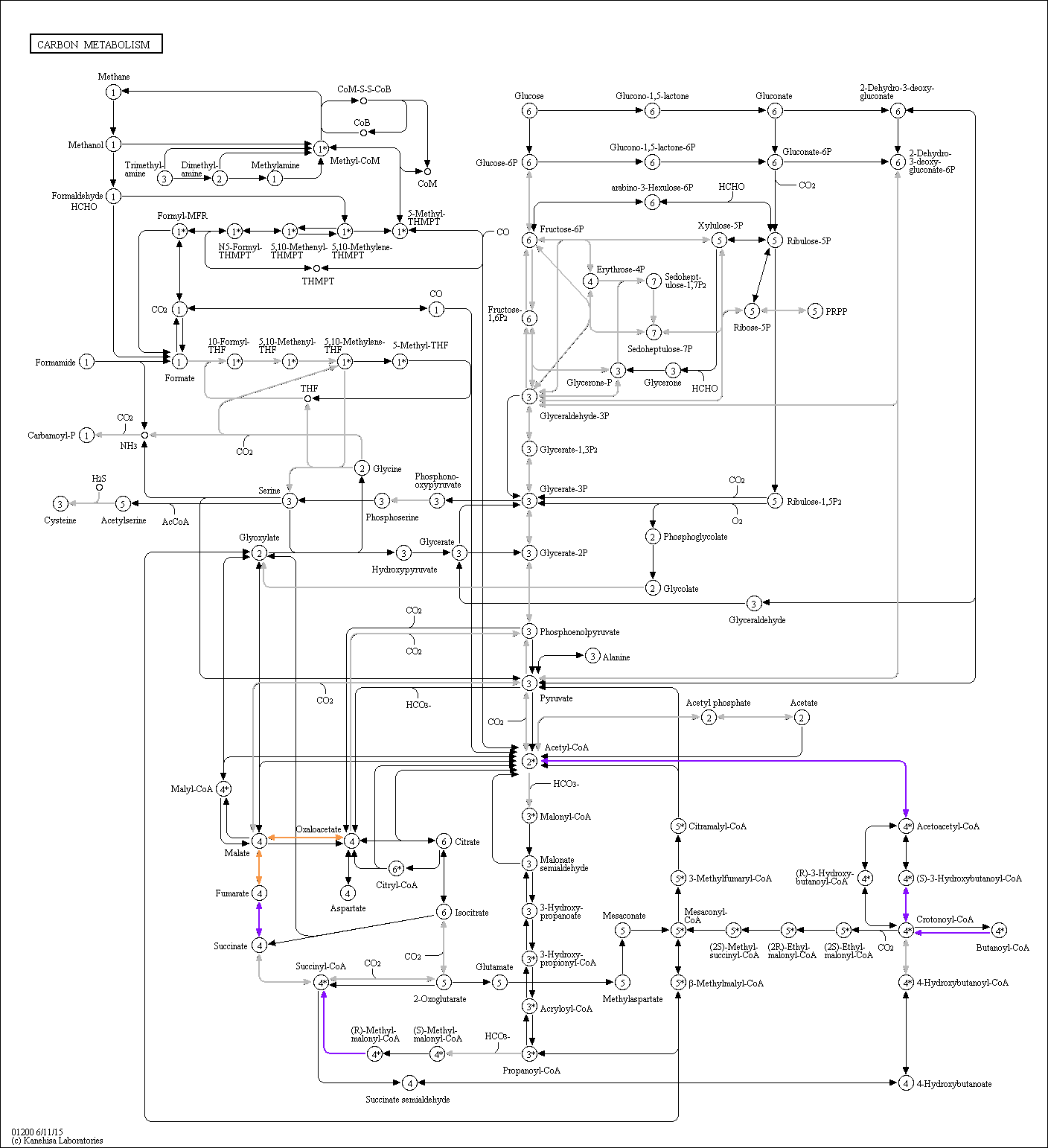

Supplement: Supplementary file 7 — Metabolic functions with differential abundance between Firmicutes and Bacteroidetes according to the MP dataset, mapped in the KEGG carbon metabolism pathway. Purple arrows indicate proteins with significantly higher abundance in Firmicutes, orange arrows indicate proteins with significantly higher abundance in Bacteroidetes, and gray arrows indicate proteins detected in one or both phyla but with no differential abundance. (PNG 38 kb) [file 40168_2017_293_MOESM7_ESM.png]
